# Ambulatory management of low-risk febrile neutropenia in adult oncological patients. Systematic review

**DOI:** 10.1007/s00520-023-08065-y

**Published:** 2023-12-13

**Authors:** Ester Forcano-Queralt, Cristina Lemes-Quintana, Domingo Orozco-Beltrán

**Affiliations:** 1 Gran Canaria Island Maternal-Infant University Hospital Complex, 35016 Las Palmas de Gran Canaria, Spain; 2 Clinical Medicine Department, School of Medicine, University Miguel Hernández de Elche, 03550 San Juan de Alicante, Spain

**Keywords:** Febrile neutropenia, Disease management, Systematic review, Quality of life

## Abstract

**Purpose:**

Recent clinical practice guidelines have recommended ambulatory management of febrile neutropenia in patients with low risk of complications. Although some centers have begun developing management protocols for these patients, there appears to be a certain reluctance to implement them in clinical practice. Our aim is to evaluate the strengths and weaknesses of this strategy according to available evidence and to propose new lines of research.

**Methods:**

Systematic review using a triple aim approach (efficacy, cost-effectiveness, and quality of life), drawing from literature in MEDLINE (PubMed), Embase, and Cochrane Library databases. The review includes studies that assess ambulatory management for efficacy, cost-efficiency, and quality of life.

**Results:**

The search yielded 27 articles that met our inclusion criteria.

**Conclusion:**

In conclusion, based on current evidence, ambulatory management of febrile neutropenia is safe, more cost-effective than inpatient care, and capable of improving quality of life in oncological patients with this complication. Ambulatory care seems to be an effective alternative to hospitalization in these patients.

**Supplementary Information:**

The online version contains supplementary material available at 10.1007/s00520-023-08065-y.

## Introduction

Neutropenia is a risk factor for certain infections that may appear as a frequent and severe complication in oncological patients; its only typical symptom is fever [1].

This entity has classically been treated through hospitalized management and intravenous (i.v.) administration of wide-spectrum antibiotics in all patients. Currently, only a fraction of patients with febrile neutropenia present serious complications, and there is evidence of a diverse prognostic spectrum [2–10]. In light of this fact, the main scientific societies at a national and international level, including the American Society for Clinical Oncology (ASCO) [11] and the Spanish Society for Medical Oncology (SEOM) [12], make a distinction between patients with febrile neutropenia who are at high or low risk, depending on defined criteria set out in globally validated scales like the ones from the Talcott group [3], the Multinational Association of Supportive Care in Cancer (MASCC) [13], and the clinical index of stable febrile neutropenia (CISNE) [14]. Better understanding of these different risk profiles, plus the emergence of new care models like home hospital care, have raised the possibility of less aggressive treatment options, for instance simplified oral monotherapy and immediate or early discharge following an observation period.

This qualitative systematic review aimed to assess ambulatory care strategies in adult patients with febrile neutropenia through a triple aim, examining efficacy, cost-effectiveness, and quality of life. These results can inform the definition of the most adequate setting for managing these patients (ambulatory care, home hospitalization, or inpatient care); contribute to updating the action protocols in emergency services; and identify areas where there is uncertainty in the available evidence in order to trace new lines of research.

## Methods

### Design and search strategy

Using the MEDLINE (PubMed), Embase, and Cochrane Library databases, a search was undertaken of all meta-analyses, clinical trials, and observational studies published up to February 2021. We also performed backward reference searching on the bibliographies of the most recent ASCO [11] and SEOM [12] clinical guidelines to look for additional studies that met our inclusion criteria.

The search strategy used the following terms:

“Febrile neutropenia,” “low risk febrile neutropenia,” and “Low risk Neutropenic fever” in three search strings, each combined with the following terms using the Boolean operator AND: “Hospital admission,” “inpatient management,” “outpatient management,” “Home admission,” “Ambulatory management,” “Early discharge,” “immediate discharge,” “delay discharge,” “Observation before discharge,” and “outpatient treatment.”

### Study selection

Eligible studies were those comparing in-hospital versus ambulatory management with immediate or early discharge, those assessing simple or combined treatment regimens, and those aiming to define the most appropriate administration route. We also included studies that analyzed the costs of different interventions or evaluated patient-reported quality of life according to the applied treatment strategy.

Studies in pediatric or adolescent populations (< 18 years of age) were excluded, as oncological pathologies have peculiarities in this group that are not generalizable to adults.

## Results

**Fig. 1:**
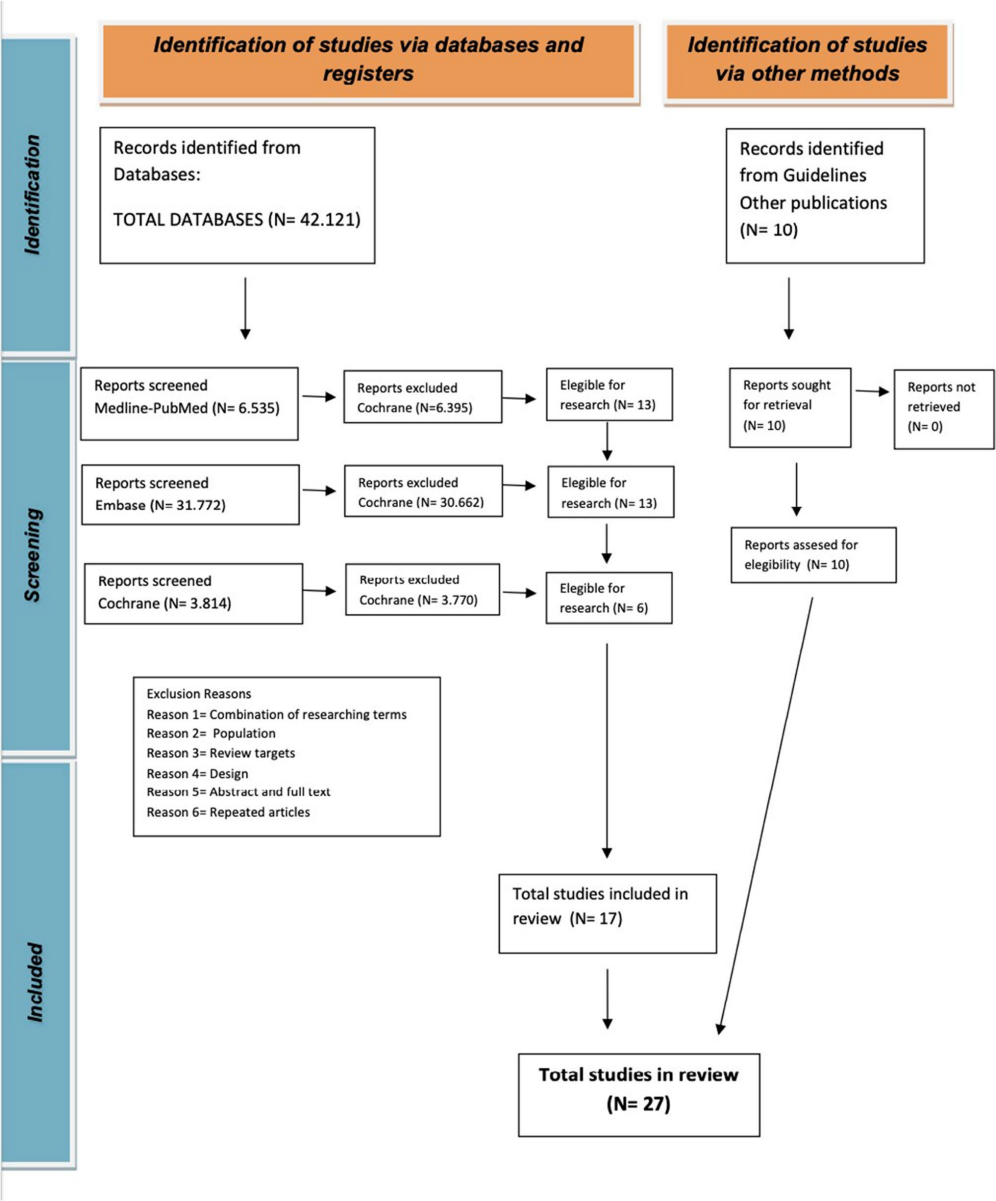
Study flow chart

The results of the search and the study flow chart are presented in Fig. 1.

### Description of included studies

The criteria for defining the concepts of fever and neutropenia in different studies are presented in annex tables 2 and 3. Included studies used heterogeneous criteria to define risk levels in their samples (Table 1).

**Table 1.** Exclusion criteria for low-risk treatment group, by organ, organ system, or medical specialty

Criteria	Studies
*Cardiovascular system*	
Heart failure	Hidalgo et al. 1999 [6]
Hypotension	Talcot et al. 2011 [3], Hidalgo et al. 1999 [5], Minoti et al. 1995 [7], Rubenstein et al. 1993 [9] (< 90 mmHg), and Mizuno et al. 2006 [15]
Taking drugs causing QT prolongation	Sotalol and dofetilide in Rolston et al. 2003 [16]
*Respiratory system*	
Tachypnea/respiratory insufficiency	Talcott et al. 2011 [3], Minotti et al. 1999 [7] and Rubenstein et al. 1993 [10] (> 30 bpm), and Mizuno et al. 2006 [15]
*Nervous system/psychiatry*	
Impaired consciousness	Talcot et al. 2011 [3], Hidalgo et al. 1999 [6]
Unfavorable psychological status	Sebban et al. 2008 [4], Rapoport et al. 1999 [8], Linaratnam et al. 2013 [17]
*Kidney*	
Acute kidney failure Oliguria/anuria	Talcott et al. 2011 [3], Rapoport et al. 1999 [8], Malik et al. 1995 [9] (dialysis); Minotti et al. 1999 [7] (eGFR < 30 ml/min); Rolston et al. 2010 [18], Rolston et al. 2003 [16], and Rubenstein et al. 1993 [10] (eGFR < 50 ml min or creatinine > 2.5 mg/dl) Hidalgo et al. 1999 [6]
*Liver*	
Liver failure	Rolston et al. 2010 [18], Rolston et al. 2003 [16], Malik et al. 1995 [9], Rubenstein et al. 1993 [10], and Hocking et al. 2012 [19] (AST, ALT > 4× normal), Sebban et al. 2008 [3] (AST, ALT > 5× normal)
*Digestive system*	
Nausea, vomiting, diarrhea	Mizuno et al. 2006 [15]
Severe mucositis	Hocking et al. 2012 [19] and Mizuno et al. 2006 [15]
Dysphagia	Talcott et al. 2011 [3], Hidalgo et al. 1999 [6], Malik et al. 1995 [9], and Poprawski et al. 2018 [20]
*Endocrine system*	
Malnutrition	Lingaratnam et al. 2013 [17] and Mizuno et al. 2006 [15]
*Metabolism*	
Acidosis	Hidalgo et al. 1999 [6]
Hyponatremia	Rubenstein et al. 1993 [10] (< 128 mg/dl)
Uncontrolled hypercalcemia	Rubenstein et al. 1993 [10]
*Hematology*	
Altered coagulation	Hidalgo et al. 1999 [6]
Prolonged neutropenia	Malik et al. 1995 [9] (> 7 d), Lingaratnam et al. 2013 [17] (> 7 d)
Use of granulocyte colony-stimulating factors	Minotti et al. 1999 [7]
Transplant patients with hematopoietic parents	Rapoport et al. 1999 [7], Minotti et al. 1999 [7], and Weyckert et al. 2014 [21]
Absolute neutrophil count ≤ 0.5 × 10^9^/L 24 h	Rapoport et al. 1999 [8]
*Infectious diseases*	
HIV	Rapoport et al. 1999 [8]
Confirmed/severe site infection	Poprawski et al. 2018 [20], Lingaratnam et al. 2013 [17], Rolston et al. 2010 [18], and Hidalgo et al. 1999 [6]
Previous use of antibiotics	14 days prior to recruitment, Rapoport et al. 1999 [8]; 5 days prior, Minotti et al. 1999 [7]; 4 days prior, Sebban et al. 2008 [4] and Kern et al. 2013 [2] (except 1 oral dose or IV in the previous 8 h); 96 h days prior, Hidalgo et al. 1999 [6] and Malik et al. 1995 [9]; and prophylactic quinolones, Goodman et al. 2017 [22]
*Gynecology*	
Pregnant or breastfeeding	All clinical trials and observational studies
*Other*	
No decrease in fever	Rapoport et al. 1999 [8] and Poprawski et al. 2018 [20]
Last cycle of chemotherapy < 12 days prior	Weycker et al. 2014 [21]
Allergy to the antibiotic used	All clinical trials and observational studies
Recurrent fever of unknown origin	Malik et al. 1995 [9]
More than one primary tumor	Weycker et al. 2014 [21]
Poor treatment adherence	Innes et al. 2003 [5]
Admitted for other reason	Hocking et al. 2012 [19]
Lack of written informed consent	All clinical trials and observational studies
Absence of caregiver	Talcott et al. 2011 (24 h) [3], Sebban et al. 2008 [4], Innes et al. 2003 [5], Hidalgo et al. 1999 [6] (24 h)
Patient refusal	Sebban et al. 2008 [4]
Distance between residence and reference center	Kern et al. 2013 (>1 h) [2]; Hidalgo et al. 1999 [6] and Talcott et al. 2011 (>2 h) [3]; Minotti et al. 1999 [7] (> 20 miles [32 km] from hospital), Rubenstein et al. 1993 [10] and Rolston et al. 2003 [16] (> 30 miles [48 km] from hospital), Lingaratnam et al. 2013 [17] (> 40 km radius)
Decline following observation period	Kern et al. 2013 [2], Sebban et al. 2008 [3], Innes et al. 2003 [5], Rapoport et al. 1999 [8], Rubenstein et al. 1993 [10]
Progression of underlying illness	Hidalgo et al. 1999 [6] and no remission in leukemia in Talcott et al. 2011 [3]
Hematological neoplasms	Lingaratnam et al. 2013 [17]
Prior episode of febrile neutropenia included in the study	Rapoport et al. 1999 [8]
Ease of obtaining educational requirements	Poprawski et al. 2018 [20]

*AST* aspartate transaminase, *ALT* alanine aminotransferase, *eGFR* estimated glomerular filtration rate

At present, there are three available scales for estimating the risk of medical complications in patients with febrile neutropenia: the rules developed by Talcott et al. [3] and the MASCC [13] (from 0 to 26 points, a score ≥ 21 is predictive of low risk) and CISNE [14] (from 0 to 8 points, with three levels of risk: I or low ≥ 2; II or intermediate ≥ 4; and III or high > 4). These validated scoring systems assume neutropenia and fever status for certain patients without regard to the severity or duration of the neutropenia as predictors of medical complications, which could require or prolong hospitalization.

Validated criteria have been used to identify patients with febrile neutropenia at low risk in clinical trials (since 2008) and observational studies (since 2010). Except for the studies by Talcott et al. [3], Mizuno et al. [15], and Rolston et al. [16], which use the Talcott criteria [3], all other clinical trials and observational studies have used the MASCC score, generally with a cutoff of 21 points to define low risk (Kern et al. [1] used a cutoff of 20 points). No included studies used the CISNE scale. However, some studies have employed clinical, biochemical, and sometimes psychosocial criteria to establish risk levels, including the studies by Rubenstein et al. [10] and Hidalgo et al. [6]. Likewise, Weycker et al. [21] also did not use a specific scale in their retrospective cohort study, instead choosing treatment arms based on the diagnostic setting.

In all included clinical trials, the ratio of patients with hematological neoplasms (leukemia or lymphoma) to solid tumors was approximately 1:4 to 1:3. All but two observational studies assessing a low-risk intervention protocol included only patients with solid tumors: the ratio in the study by Poprawski et al. [20] was 1:3, while the distribution between tumor types in the study by Goodman et al. [22] was roughly equal.

Antibiotic treatments used, administration routes, and treatment settings are presented in annex tables 2 and 3. Moreover, the samples of almost all clinical trials and observational studies included 25 to 38% of people being treated with colony-stimulating factors (CSFs), except for Innes et al. [5], who excluded them.

Between 6 and 20% of patients treated on an ambulatory basis were readmitted.

### Analysis of efficacy

As for the results of the efficacy analysis, Rubenstein et al. [9], Minotti et al. [6], and Sebban et al. [4] did not find differences between oral versus intravenous administration in low-risk patients with neutropenia. The latter two studies evaluated a monotherapy antibiotic regimen. Malik et al. [9] found that oral fluoroquinolone monotherapy was equally effective whether administered in hospitals or other settings. Rapoport et al. [8] likewise obtained similar results in patients receiving classical inpatient care or early discharge with ambulatory therapeutic management. In line with these results, Hidalgo et al. [6] did not find differences in treatment response between an inpatient i.v. double regimen versus oral monotherapy, administered in ambulatory care following good response to the first dose in hospital and early discharge. These findings were also reproduced by Innes et al. in 2003 [5], who compared double i.v. therapy in-hospital versus double oral treatment in ambulatory care. For their part, Talcott et al. [3] did not observe a higher rate of complications using an ambulatory strategy. Finally, Kern et al. [2] reported similar results between double versus monotherapy in the ambulatory setting.

In the observational studies, Mizuno et al. [15] suggested that telephone follow-up could be an effective strategy to support ambulatory management. Cooksley et al. [23] drew similar conclusions, speculating that ambulatory treatment could be more feasible if clinical oncological support were available; this group provided such support in the form of an oncologist on call through a 24 h hotline. Rolston et al. [16] evaluated an early discharge strategy with gatifloxacin monotherapy 400 mg versus moxifloxacin 400 mg, suggesting that quinolone monotherapy could be a safe and effective intervention in low-risk patients selected using the Talcott criterio [3] and the MASCC score, respectively. In addition, Hocking et al. [19] implemented an ambulatory management strategy aligned with Australian practice guidelines in 2012, concluding that this could be a valid strategy for selected patients with solid tumors and low-risk febrile neutropenia. In their retrospective analysis, Weycker et al. [21] obtained similar results in a sample of patients with mostly solid tumors, a small proportion of whom were treated exclusively as outpatients. Goodman et al. in 2017 [22] and Poprawski in 2018 [20] also reached the same conclusion based on their studies, with ratios of 1:1 and 1:3, respectively, of hematological to solid tumors. Goodman et al. [22] noted that applying social and non-medical criteria in addition to the MASCC score complicated ambulatory treatment approaches in low-risk patients with febrile neutropenia. In the same line as Hocking et al. [19], Lingaratnam et al. [17] analyzed the successful implementation of the Australian protocol in a center in Victoria. Even though there was a sizable proportion of patients who could not initially be classified as low risk, their evolution permitted a change to the oral treatment group, and none presented treatment failure.

### Cost-effectiveness analysis

Our search strategy yielded a total of 8 articles with a focus on cost-effectiveness: 1 clinical trial, 2 cohort studies, and 5 other observational studies. The characteristics of the studies (research group, year of publication, country, definition of febrile neutropenia, mean age, design, perspective, source of economic data, length of hospital stay, direct and indirect costs, and summary of results) are shown in annex table 4.

This analysis did not use data from other included studies that mentioned cost savings without providing a detailed explanation of the methods used to calculate them, as the 8 studies that are included in this analysis did.

Three studies analyzed the direct costs derived from inpatient management of febrile neutropenia. Kuderer et al. [24] reported that the health care expenditure from each episode of febrile neutropenia arose mainly from the costs of the hospital stay along with associated infections and comorbidities. Mayordomo et al. [25] went into more detail, itemizing the costs for each classical intervention and showing that hospitalization accounted for 79% of the costs, followed by antibiotic treatment (10%), CSF (5%), complementary tests (4%), and transfusions (1%). Larger costs were incurred (in-hospital stay, interventions, and complementary tests) in patients with lymphoma compared to breast or lung cancers. Finally, O’Brien et al. [26] added that health care costs in patients aged 65 years or older were significantly higher.

Five included studies assessed the economic impact of an ambulatory-based treatment strategy. In 2000, Elting et al. [27] found that an ambulatory strategy incurred lower costs than i.v. treatment during admission. In their 2008 cohort [28], the same group found no differences in the efficacy of hospital versus ambulatory treatment, and even though just 21% of the ambulatory group were readmitted, the cost in that arm was double. In 2011, Hendricks et al. [29] reported savings of USD 5354 using an ambulatory strategy with early discharge, considering the costs derived from the initial emergency department consultation, complementary studies, home interventions, pharmacological treatment, and daily nurse home visits. In 2017, Teh et al. [30] reported 2.65 times lower costs after applying an ambulatory strategy in low-risk patients compared to a comparable historic cohort in the same hospital. Finally, Borget et al. [31] aimed to identify where the largest economic burden came from, comparing the standard hospital strategy versus outpatient treatment in hospital and ambulatory treatment outside of it, reporting successive decreases in costs in each setting.

### Analysis of patient-reported quality of life

Patients’ perceived health was only specifically analyzed in relation to ambulatory treatment strategies by Teuffel et al. in 2011 [32], who asked cancer patients to hypothetically consider what their preferred treatment strategy would be if they presented febrile neutropenia during the course of their disease. Authors observed that patients generally preferred early discharge plus ambulatory treatment with oral rather than i.v. administration. Patients were willing to renounce up to 10 weeks of their life and pay between USD 255 and USD 327 more if they could avoid hospitalization. However, in individual interviews, some perceptions changed substantially. Details of this research are presented in annex table 5.

In addition, two of the included clinical trials—one by the Talcott group in 2011 and another by Sebban et al. in 2008 [4]—performed a simple quality of life analysis using two questionnaires. The first applied the European Organization for Research and Treatment of Cancer quality of life questionnaire (EORTC QLQ-C30), a validated, self-administered instrument, finding a significant reduction in the perception of pain in patients from the ambulatory treatment arm. The second applied a quality of life questionnaire 24 h after finalizing treatment, finding no differences between intervention groups.

## Discussion

Since at least the early 1990s, with Rubenstein et al. [10], and possibly even before then, there has been recognition that the risk of complications is heterogeneous in populations with febrile neutropenia. The few studies available on this topic have been characterized by the diversity of their aims, approaches, and patient selection criteria. Factors contributing to this heterogeneity include evolving knowledge on the entity; the emergence of new oncological treatments (chemotherapies, CSFs, antibiotics); the growing tendency to simplify treatments to favor adherence and tolerability and to reduce adverse effects; and the decentralization of health care away from hospitals due to associated comorbidity, impaired quality of life, and increased cost.

Indeed, even the definition of fever itself and the best place to measure it have changed several times since Carl Wunderlich’s early work on it in 1868. This branching evolution is evident in the current definitions of the Infectious Diseases Society of America and other societies in South America, Europe, and Asia, and in the different ways that our included studies identify it; however, it is not necessarily reflected in the publication year of our included studies.

These changes are also reflected in the fact that Minotti et al. [7] excluded patients with neutrophil counts under 300/μL because at that time, severe neutropenia, especially with neutrophil counts of less than 100/μL, was considered a marker of high risk. Later studies do not reflect the application of this criterion. Indeed, the interpretation of our results must consider the variations in the neutrophil count of included and excluded patients. Kern et al. [1], Sebban et al. [4], Innes et al. [5], Hidalgo et al. [6], and Malik et al. [9] all included patients with moderate neutropenia (500–1000/μL) with the potential to become severe neutropenia (< 500/μL). However, only Malik et al. [9] clarified that the patients whose neutrophil counts did not drop below the 500/μL threshold were excluded. However, given the inclusion of patients with critical levels of neutropenia in other studies, this distinction may be irrelevant.

Another fact suggestive of the diversity of selection criteria is that despite criticism of Talcott’s group and the MASCC and CISNE scales for not considering the neutrophil count as a criterion for risk assessment, the risk associated with this aspect does not seem to determine the appropriateness of managing neutropenia on an ambulatory basis. Indeed, good outcomes were achieved with simplified therapies in the ambulatory setting regardless of the criteria for selecting candidates. In the studies that did report the proportion of patients with neutrophil counts that dipped below 100/μL (Kern et al. [2], Talcott et al. [3], Hidalgo et al. [6], Minotti et al. [7], Malik et al. [9], and Rubenstein et al. [10]), these proportions were described as similar between groups in all studies but Rubenstein et al.’s, who did not specify.

Regarding the risk assessment scales, the ASCO 2018 [11] guidelines recommend the use of MASCC followed by CISNE, while the SEOM 2018 [12] guidelines prefer only the CISNE. However, the included studies did not evaluate the latter scale as a method to identify candidates for ambulatory care. Moreover, CISNE has not been validated in hematological patients, restricting the subgroups eligible for less aggressive interventions. Furthermore, Ahn et al. [33] demonstrated that the MASCC had greater discriminatory power than the CISNE for detecting low-risk patients, with greater sensitivity and negative predictive value, resulting in a smaller likelihood of including patients at true high risk of complications in ambulatory regimes. Thus, in 2013, Ahn et al. [34] already proposed procalcitonin values as an additional parameter for the MASCC to refine the probability of presenting bacteremia or septic shock, in patients at both low and high risk. Even within the latter group, the authors recognized heterogeneous prognoses, calling into question the need for indiscriminately applying aggressive management.

The evidence on antibiotic treatment approaches, both for double therapy with amoxycillin/clavulanic acid (or clindamycin in patients with allergies) plus quinolone and for monotherapy with fluoroquinolone, shows good efficacy outcomes in and outside of the hospital setting (Kern et al. [2], Sebban et al. [4], Hidalgo et al. [6], Minotti et al. [7], Malik et al. [9]). Despite these results, the ASCO guidelines [11] continue advising against fluoroquinolone monotherapy for ambulatory management of low-risk patients, while SEOM affirms that the only alternative in penicillin-allergic patients is hospital admission and i.v. treatment.

In 20 to 30% of febrile neutropenia cases, the germs responsible are identified, predominantly gram-positive rather than gram-negative bacteria, followed by polymicrobial and fungal infections (Malik et al. [9], Rapoport et al. [8], Hidalgo et al. [6], Minotti et al. [7], Sebban et al. [4]). These results are consistent with other available studies in the literature.

With respect to the use of CSFs, it has been known for decades, as reported by studies like Mather et al. in 1994 [35], that these interventions are capable of reducing the mean duration of the neutropenia by 1 day as well as the total time needed for resolution, although not the total days of fever. No study has explained how authors decided to administer this treatment, describing the criteria, type of neoplasm, level of neutrophils, or timing of administration (from the last session of chemotherapy or emergency presentation). Similarly, the literature has not explored how CSFs may have influenced the outcome (patient recovery or the success of the treatment strategy). There are also gaps in research investigating whether prescribing CSFs would result in savings given the implicit pharmacological costs associated with hospitalizing these patients in a hospital or their home, and whether it would be possible to reduce the duration of antibiotic therapy in patients with a fever of unknown origin and negative culture.

Several strategies for ambulatory follow-up have been evaluated, including home visits from health professionals, hotlines, scheduled telephone calls, and outpatient hospital visits. However, no studies have compared these interventions with each other to assess treatment response, adverse effects, cost-efficiency, or quality of life. These aspects could be the focus of future lines of research.

Although teenagers were not included as population in our systematic review, because we excluded studies with population under 19 years old in our criteria research, in many countries, they are treated around 16 years old in emergency departments as adults. Authors, such as Klastersky et al. [36], carried on an early discharge strategy after 24 h, til 48 h in many of the episodes of febrile neutropenia in people that includeed 16 years old teenagers [36].

Their results show that we could safely proceed in low-risk teenagers according to MASK score, in the same way as adults with an oral combination therapy of peniciline and quinolones, if not allergic, with a proposed followed up by phone, temperature control each/6 h, and blood control test each 48 h until accomplishing criteria of recovering. We should highlight in this investigation team, they first give a definition more than absence of hospital readmission as a criteria of succes of oral empirical therapy as 5 days without fever, no clinic symptoms, and signs of infection and pathogen erradication.

Within the sphere of ambulatory management, four studies proposed that patients receive hemograms every 2 to 3 days after discharge, at least until achieving neutrophil counts of 1000/μL, indicating mild neutropenia (Goodman et al. [22], Kern et al. [2], Sebban et al. [4], Hocking et al. [19]). Although several studies have evaluated the cost of ambulatory follow-up, there has been no calculation of the specific cost of such controls, which could generate substantial amounts of research information. It may be appropriate to restrict these control visits to cases with poor evolution or to limit them to one visit at the end of treatment.

Another strategy that could be analyzed for its efficacy and cost-efficiency would be the performance of a preliminary assessment of oncological patients’ household situation (including family support) by home hospital team, prior to initiating chemotherapy. This assessment could help facilitate decision-making in emergency departments around managing these patients.

Many studies have limited ambulatory approaches to patients who live at some distance from the reference center or hospital (Kern et al. [2], Hidalgo et al. [6], Talcott et al. [3], Minotti et al. [7], Rubenstein et al. [10], Rolston et al. [18], Lingaratnam et al. [17]). To reduce the population of low-risk patients excluded from this strategy, future research could also assess the efficacy, patient satisfaction, and cost-efficiency associated with strategies in coordination with primary health care teams, for patients who live far from the hospital and whose only limitation for inclusion in an ambulatory regime is distance.

Available studies suggest than an ambulatory strategy for low-risk patients with febrile neutropenia is cost-efficient as well as effective. This is true for interventions assessed both in clinical trials (Hendricks et al. [29], Elting et al. [27]) and in prospective studies, for example, in those evaluating the implementation of new protocols compared to data from historical cohorts fulfilling the same criteria (Teh et al. [30], Mayordomo et al. [25]). Economic studies should be considered in light of potential differences in the source of economic data and their perspective. Establishing the most appropriate design to allow comparison with other studies can be complicated because health systems are not managed the same across different countries, nor is access to different therapeutic options the same in privately managed systems. Thus, in future studies, it will be necessary to establish quality criteria in order to favor reliability and comparability of the economic data obtained.

With regard to quality of life, Teuffel et al. [37] suggested that patients prefer ambulatory strategies, although data did not show differences in the short quality of life evaluations performed in two clinical trials in people with febrile neutropenia. In any case, more structured trials that specifically aim to assess this domain are needed before drawing any firm conclusions.

Despite the positive evidence, fewer patients benefit from ambulatory strategies than expected (for clinical, socioeconomic, and geographic reasons). Health services still seem reluctant to implement the action protocols in low-risk patients that are recommended by national and international clinical practice guidelines, even though these tightly restrict the application of ambulatory models.

Available evidence on efficacy, quality of life, and cost-efficiency seems to support ambulatory approaches for low-risk patients with febrile neutropenia. The main societies and services involved in managing these patients should establish more standardized priorities and criteria to guide future research and reach more definitive conclusions. Studies are needed to analyze the facillitators and barriers for the instigation and maintenance of an ambulatory febrile neutropenia management programmes.

### Supplementary information

ESM 1 (PDF 344 kb)

## Data Availability

N/A.

## Code availability

N/A.
