# Supplementary material for: Ambulatory management of low-risk febrile neutropenia in adult oncological patients. Systematic review
Source: Support Care Cancer. 2023 Dec 13;31(11):665. doi: 10.1007/s00520-023-08065-y (PMC10624743; doi:10.1007/s00520-023-08065-y)
Supplement: Supplementary file 1 — (PDF 344 kb) [file 520_2023_8065_MOESM1_ESM.pdf]

Annex. Table 2. Analysis of efficacy. Clinical trials.

Author	Population ST/L-L	Mean age	Criteria for low-risk	Febrile neutropenia definition	Discharge	Inpatient management	Days of treatment	Outpatient management	Days of treatment	Hospital readmission	Fever of unknown origin	ANC <100/μL
Kern, 2013	49/51<br><br>49/51	50<br><br>49	MASCC >20	Oral or timpanic temperature > 38.5°C ò ≥ 38°C 2 times measurement separated by 1 hour, in a period of 12 h<br><br>ANC< 1000 with an expected decrease <500 in 24h.	3h	-	-	Oral Moxifloxacin 400mg/24h<br>v.s.<br>Ciprofloxacin 750mg + oral Amoxi/Clav 1g/12h	7d<br><br>7d	16%<br><br>15%	56%<br><br>62%	52%<br><br>43%
Talcott, 2011	71/29	47	Risk assesement criteria of Talcott et al	T≥100.5ºF<br>ANC <500 ≥24h	-	Not specified<br><br>Penicilin + aminoglycoside or ceftazidime<br><br>Imipenem<br>Aztreonam<br>Aminoglycoside + vancomycin<br><br>CSF (38%)	-	Not specified<br><br>Penicilin + aminoglycoside or ceftazidime<br><br>Imipenem<br>Aztreonam<br>Aminoglycoside + vancomycin<br><br>CSF (37%)	-	9%	-	Median ANC 57/μL (range 0-980) (IM)<br><br>Median ANC 100 /μL (range 0-600) (OM)
Sebban, 2008	70/30	52	MASSC≥21	Oral T ≥38,5ºC or ≥38ºC 2 times in 12h<br><br>ANC<500 or <1000 with expected decline to <500	24-48h	-	-	IV Ceftriaxone 2gr/24h<br><br>Oral Moxifloxacin 400mg/24h	5d<br><br>4d	14,9% in both	71%	-
Innes, 2003	69/31	50-53	Clinical, biochemical and psychosocial aspects	Oral T ≥38ºC 2 times every 4h in 24h<br><br>ANC<500 or <1000 with expected decline to <500	24h	Piperacillin tazobactam (4g/500mg) and IV Gentamicin 80mg/8h	-	Ciprofloxacin 750mg/12 + Oral Amoxi/Clav 500mg/125mg/8h	-	-	37%	-
Hidalgo, 1999	89/11	55-56	Clinical, biochemical and psychosocial	Axillary T >38ºC<br><br>ANC <1000 with expected decline to 500-1000 or < 500	Immediate	IV Ceftazidime 2gr/8h<br>+<br>IV Amikacin 500mg/12	-	Oral Ofloxacin 400mg/12h	-	8/48 (16.6%)	68%	41%
Minnotti, 1999	54/46	59-60	Clinical, and biochemical	T>38ºC 2 times < 24h<br>ANC not <300	Immediate		-	IV Ceftriaxone 1g/24h<br><br>Oral Ciprofloxacin 750mg/12h	5-10d	17/93	-	Exclusion ANC <300

Rappoport, 1999	70/30	45-48	Clinical, and biochemical	Axillary T ≥38,5ºC or > 38ºC repeated, ANC <500	48-72h	Ceftriaxone 2gr/24h x5 d + aminoglycoside (gentamicin + netilmicin 4.5-6.5 mg/kg, 300mg max or amikacin 20mg/kg, 1,5 gr max) ≥2 d<br><br>CSF until ANC≥1000	5d	Ceftriaxon 2gr/24h x 5d + aminoglycoside (gentamicin + netilmicin 4.5-6.5mg/kg, 300mg max or amikacin 20mg/kg, 1,5 gr max) ≥2 d<br><br>CSF until ANC≥1000	5d	-	OM= 66%<br>IM=57%	36%
Malik, 1995	69/31	43-46	Clinical, biochemical and psychosocial	Oral T ≥38,5ºC or >38ºC 2 times in 4 h ANC<500 or <1000 expected decline to <500 in 24h	Immediate	Oral Ofloxacin 400mg/12h	-	Oral Ofloxacin 400mg/12h	-	21%	71%	49%
Rubenstein, 1993	74/26	59-60	Clinical, biochemical and psychosocial	-	8h	ASCORP-I<br>Aztreonam + IV Clindamicyn<br><br>ASCORP-II<br>Aztreonam + IV Clindamicyn	8d	ASCORP-I<br>Ciprofloxacin 750mg/12h + Oral Clindamicyn<br><br>ASCORP-II<br>Ciprofloxacin 500mg/12h + Oral Clindamicyn	7d	6/83	61%	59%

ANC: Absolute neutrophil count (cel/µL), T: Temperature, CSF: colony-stimulating factors, IM: Inpatient management, OM: Outpatient management, Amoxi/Clav: Amoxicilin/Clavulanic, IV: intravenous. ST: Solid tumor, L-L: Lymhoma-Leukemia.

Annex. Table 3. Analysis of efficacy: Observational studies

Author	Design	Population TS/L-L	Mean age	Criteria for low-risk	Febrile neutropenia	Inpatient management	Outpatient management	Median hospital stay	ANC <100 (%)
Poprawski, 2018	Retrospective	60,7/39,3	65	MASSC≥21	T≥38ºC<br>ANC<1000	IV CSF	CSF	Low risk 3 days (0-15)<br>* Early discharge 24h, stay 1,5 days<br>High risk 5 days (0-13)	-
Cooksley, 2018	Prospective	94,1/5,9	51	MASSC≥21<br>NEWS≤3	T≥38ºC<br>ANC<1000	IV Meropenem (1st dose)	Amoxi/Clav 500/125 mg and ciprofloxacin 500mg<br><br>In case of allergy: Moxifloxacin 400 mg/24h	0.73 days	-
Goodman, 2017	Prospective	50.55/49,45	H.R.58.2<br>L.R.51.6	MASSC≥21	Oral T ≥ 38,3ºC or > 38ºC >1h<br>ANC <500	High Risk patients intravenous treatment	Amoxi/Clav 875/125mg/12h + ciprofloxacin 750mg/12h<br><br>ciprofloxacin 750mg/12h + clindamicyn 600mg/8h	High risk = 6,5 days<br>Low risk = 3,7 days<br><br>Low risk ≤48 h	-
Lingaratnan, 2013	Prospective	-	-	MASSC≥21	Oral T ≥ 38,3ºC or > 38ºC 2 times.<br>ANC <500 or < 1000 expected decline to <500	IV and early oral.<br><br>Amoxi/Clav 875/125mg/ 12h and/or ciprofloxacin 500-750mg/12h	Oral dual therapy<br>Amoxi/Clav 875/125mg/12h + ciprofloxacin 500-750 mg/12h	Low Risk = 5-6 days<br>High Risk = 7-13 days	-
Weycker, 2013	Retrospective	89,3/10.7		based on the initial setting of care (emergency department vs intensive care unit)	ANC <1000 or presence of infection or fever (T≥38,3ºC)	-	-	8,4 days in inpatient management<br><br>Outpatient encounters: 69.2% in the physician's office, and 26.9% in the emergency department.	-
Hocking, 2012	Prospective	100/0	-	MASSC≥21	T>38ºC<br>ANC < 1000	-	Amoxi/Clav 875/125mg/12h + ciprofloxacin 500mg/12h	Low Risk<br>- Inpatient 6,5 days<br>- Early discharge 2,2 days	-
Rolston, 2010	Prospective	100/0	44	MASSC≥21	T ≥38,3ºC<br>ANC <500 or < 1000 expected decline to <500	Moxifloxacin 400mg/24h (1st dose)	Moxifloxacin 400 mg/24h	Early discharge after 4-8h observation	76%
Rolston, 2003	Prospective	100/0	44	Talcott et all<br>MASCC	T ≥38,3ºC<br>ANC <500 or < 1000 expected decline to <500	Gatifloxacin 400mg/24h (1st dose)<br>73% CSF	Gatifloxacin 400mg/24h	Early discharge after 4-8h observation	80%
Mizuno, 2006	Retrospective	-	50.5	Talcott et all	Oral or axillary T > 38ºC 8-14 days after initiating chemo. Expected duration of ANC <500 less than 10 days	Ceftazidime or ceftazidime + IV amikacin	Oral Ciprofloxacin 400mg/8h x 5 days	-	-

H.R.: High Risk, L.R.: Low Risk, Amoxi/Clav: Amoxicilin/Clavulanic, ST: Solid tumor, L-L: Lymhoma-Leukemia.

Annex. Table 4. Cost-effectiveness analysis

Author	Country	Design	Febrile neutropenia	Median age (years)	Management	Source of economic data	Length of stay (days)	Direct costs	Results
Teh, 2017	Australia	Prospective	T>38,3ºC or 38ºC 2 times<br>ANC< 1000<br>MASSC score≥21	50	Inpatient<br>Outpatient	Hospital records	1.1-4	Admission<br>Outpatient program<br>Health professionals	Less cost with an outpatient program
Borget, 2013	France	Prospective	T>38,3ºC or 38º >1h<br>ANC<500<br>MASSC score≥21	53±16	Inpatient<br><br>Inpatient with oral treatment<br><br>Outpatient	Hospital records	Outpatient: <24h<br><br>Early discharge: 4,5±3<br><br>Inpatient : 7,7±5.3	Admission<br>Pharmacological therapies<br>Complementary exams	Outpatient 576±651€<br>Early discharge 3924±1973€<br>Inpatient 6807±3673€
O'Brien, 2013	Ireland	Prospective	T>38ºC<br>ANC< 1000	58.8±10.1	Inpatient	University teaching hospital<br>Irish Casemix Programme<br>Nathional Health costs<br>Hospital pharmacy department within a University Teaching Hospital, Dublin<br>The National Blood Centre, Dublin	7	Admission<br>Pharmacological therapies<br>Complementary exams	≥65 years 10.844±1329 €<br><65 years 8428±816€ (p<0.01)<br><br>Women 8757±1007 €<br>Men 9103±9954 €<br>Lymphoma 9068±2068 €<br>Not haematological malignancy 8824±734 €<br>After 1st cycle CT 8836±712 €<br>After several cycles 9232±1109 €
Hendriks, 2011	USA	Clinical Trial	T 100.5 ºF<br>ANC <500<br>(Talcott et all)	46-47	Inpatient<br><br>Early discharge	Hospital records and Patient registers	Not specified	Admission<br>Pharmacological therapies<br>Complementary exams	Early discharge (doctor's appointment and complementary exams) 3.349 $<br>Hospital admission costs/day 2.023 $<br>Home management/day (drugs, daily visits by ambulatory care nurses) 787 $<br>Total inpatient costs 16.341 $<br>Total home management costs 10.977 $ (P<0.01)
Elting, 2008	USA	Prospective	T 38,3ºC<br>ANC< 1000<br>MASSC score≥21	50	Inpatient<br><br>Inpatient with oral treatment	Hospital records	Inpatient: 5,7<br><br>Early discharge: 1.3	Admission<br>Pharmacological therapies<br>Complementary exams<br>Health professionals	Inpatient management 15.231 $<br>Outpatient management 7.799 $ (P<0.01)

Mayordomo, 2009	Spain	Retrospective	T 38,2ºC + ANC< 500<br>T 38,5ºC + ANC< 1000	60±10	Inpatient	Hospital records	8±6.9	Admission<br>Pharmacological therapies<br>Complementary exams	79% admission 3841€<br>10% antibiotics 403€<br>> 1% other treatments<br>5% CSF<br>4% complementary exams<br>1% transfusions<br>Lymphoma 4554€ > breast and lung cancer (p<0,05)
Kuderer, 2002	USA	Prospective	Not specified *1	53.6	Inpatient	Hospital records and Patient registers	11.5- 8.1 Solid Tumor<br>10.7 Lymphoma<br>19.7 Leukemia	Admission	Cost of each episode 19.110 $
Elting, 2000	USA	Analysis review concerning 6 clinical trials in Texas from 1981-1994	-	-	Inpatient		Inpatient related with antibiotic regimen<br>A: 9d<br>B: 12d<br>A+V: 9d<br>B+V: 13d	-	Inpatient cost per day 1.002 $<br><br>Home health costs per day for IV drugs 133 $<br><br>Therapeutic regimen cost A: 8.491 $<br>Therapeutic regimen cost B: 11.133 $<br>Therapeutic regimen cost A+V: 9.952 $<br>Therapeutic regimen cost B+V: 11.412 $

Therapeutic regimen A: Imipenem, B: Ceftazidime, V: Vancomycin. *1: Limited by the nonspecific nature of the ICD-9-DM diagnostic code of 288.0 for agranulocytosis. Buck CJ. ICD-9-CM official guidelines for coding and report-ing. In: 2005 ICD-9-CM and HCPCS. Philadelphia: Saunders,2005:3-29. CT: chemotherapy

Annex. Table 5. Analysis of patient-reported quality of life

Author	Population ST/L-L	Age	Design	Methods
Teuffel, 2012	77 /23	Median 54 years (20-88)	Descriptive Cualitative	Interview<br><br>Measurement tools<br><br>-VAS score (Quality of life)<br><br>-WTP (Money willing to pay)<br>-TTO (Quality of life time willing to give up)<br>Hypothetical situations<br>1. Hospital IV treatment<br>2. Treatment at home after an initial observation with IV treatment<br>3. IV treatment at home<br>4. Oral antibiotics at home
Talcott, 2011	71/29	Median 47 years	Clinical trial	EORTC QOL-C30 (self-administered)<br><br>Other questionnaires/Subscales: GWS (General State), global assessment of quality of life. Technical scales, quality, communication, interpersonal care and consumer satisfaction scales of the American Health Association; also a study of a specific measure developed in a pilot study to assess the effects on the place of treatment, including the relationship with family members and health professionals and their personal sense of security.
Sebban, 2008	70/30	Median 47 years	Clinical trial	QOL-data after 24h of the end of the treatment

IV: intravenous, QoL: Quality of life.
